# Acute anti-proliferative and anti-migratory effects of cannabidiol on C6 rat glioma, SH-SY5Y human neuroblastoma, and HT22 mouse hippocampal neuronal cell cultures

**DOI:** 10.3389/ftox.2026.1727831

**Published:** 2026-04-21

**Authors:** Kittikun Viwatpinyo, Sujira Mukda, Aktsar Roskiana Ahmad, Sakan Warinhomhoun

**Affiliations:** 1 School of Medicine, Walailak University, Nakorn Si Thammarat, Thailand; 2 Herbology Research Center, Walailak University, Nakhon Si Thammarat, Thailand; 3 Research Center for Neuroscience, Institute of Molecular Biosciences, Mahidol University, Nakorn Pathom, Thailand; 4 Department of Pharmacognosy and Phytochemistry, Faculty of Pharmacy, Universitas of Muslim Indonesia, Makassar, South Sulawesi, Indonesia; 5 College of Oriental Medicine, Rangsit University, Pathum Thani, Thailand

**Keywords:** apoptosis, cannabidiol, cytotoxicity, glioma, hippocampal neuron, neuroblastoma

## Abstract

**Background:**

The treatment of central nervous system tumors remains challenging owing to their highly proliferative nature, aggressiveness, and poor prognosis. Additionally, existing treatment methods have several problems, including high risk of complications, systemic side effects, and impact on patients’ quality of life. Recently, cannabidiol (CBD), a non-psychoactive cannabinoid found in *Cannabis sativa*, has emerged as an alternative therapeutic medication because of its potential antitumor activity with fewer side effects.

**Methods:**

We evaluated the cell viability, clonogenicity, migration, apoptotic nuclear morphology, and cell cycle phases of C6 rat glioma, SH-SY5Y human neuroblastoma, and HT22 immortalized mouse hippocampus neuronal cultures treated with CBD ranged between 0 and 10 μg/mL.

**Results:**

CBD concentrations exceeding 5 μg/mL induced significant reductions in cell viability in C6 glioma and SH-SY5Y neuroblastoma cultures, accompanied by decreased clonogenicity in both cultures at 10 μg/mL. A scratch assay for cell migration revealed that 5 μg/mL CBD suppressed C6 glioma cell migration. Additionally, late apoptotic nuclear morphology was observed in C6 glioma cultures treated with 10 μg/mL cannabidiol. Similarly, HT22 hippocampal neuronal cultures exhibited decreased cell viability and clonogenicity, with apparent nuclear signs of apoptosis at CBD concentrations over 5 μg/mL. Notably, CBD disrupted HT22 cell migration at concentrations of 2.5 and 5 μg/mL. Proteomic profiling of C6 glioma revealed upregulation of ribosomal proteins, molecular chaperones, and modulators of cytoskeletal dynamics upon treatment with 1 μg/mL CBD. In comparison, treatment with 2.5 μg/mL CBD led to marked downregulation of endoplasmic reticulum chaperones, mitochondrial ATP synthase, and cytoskeletal regulators.

**Conclusion:**

Our findings confirm the sensitivity of glioma, neuroblastoma, and hippocampal neuronal cultures to CBD, providing valuable insights for further research into its therapeutic potential against glioma, neuroblastoma, and neuronal disorders.

## 1 Introduction

Brain and central nervous system (CNS) tumors remain among the most lethal cancer types globally despite their relatively low incidence. According to the latest GLOBOCAN 2022 estimates, approximately 322,000 new cases of brain and CNS tumors were diagnosed worldwide in 2022, with an age-standardized incidence rate of approximately 3.5 per 100,000 and nearly 248,000 associated deaths (Kim et al., 2025). These figures reflect a continued global burden and underscore the urgent need for improved therapeutic approaches, particularly for aggressive subtypes such as glioblastoma. Recent trends also suggest notable regional and demographic variations in incidence and outcomes, with higher rates observed in parts of North America and Western Europe (Filho et al., 2025). The most common type of primary malignant tumor of the CNS is a glioma, which arises from glial cells. Nearly fifty percent of all newly diagnosed gliomas in the US are glioblastomas, the most severe type of glioma with a less than 5% 5-year survival rate (Low et al., 2022; Miller et al., 2021; Ostrom et al., 2014). This poor prognosis may be due to multiple cellular factors, including high proliferation and invasion, angiogenesis induction, and the presence of glioma stem-like cells within the tumor mass (Dumitru et al., 2018; Liebelt et al., 2016), which contribute to the frequent recurrence of malignant tumor cells and their resistance to conventional therapeutic methods. Another well-known tumor of CNS is neuroblastoma, which is the most frequent type of brain and central nervous system tumor in infants as well as young children. Even though neuroblastoma has a better prognosis than glioblastoma, the co-administration of chemotherapy and radiotherapy usually leaves patients with neurological conditions such as weakness or prolonged pain sensation (Matthay et al., 2016; Park et al., 2010). Neurosurgical intervention, chemotherapy, and radiation therapy are the main therapeutic approaches for CNS malignancies currently (Omuro and DeAngelis, 2013; Wen and Kesari, 2008). In recent years, phytochemicals have gained substantial attention as alternative or integrative therapeutic agents due to their ability to modulate multiple oncogenic pathways while generally exhibiting lower systemic toxicity compared with conventional chemotherapeutic agents. Several comprehensive reviews have highlighted the potential of plant-derived compounds in hepatocellular carcinoma, breast cancer, and head and neck cancer, where phytochemicals demonstrate anti-proliferative, pro-apoptotic, anti-angiogenic, and anti-metastatic effects through regulation of oxidative stress, cell cycle checkpoints, and survival signaling pathways (Rodriguez et al., 2021; Santiago et al., 2025; Wali et al., 2025). Unlike many cytotoxic chemotherapies that are associated with severe adverse effects such as myelosuppression, gastrointestinal toxicity, neurotoxicity, and off-target tissue damage, phytochemicals are often reported to exert more selective biological activity with comparatively fewer undesirable systemic complications, although dose optimization and safety evaluation remain essential.

Owing to its antiproliferative and anti-invasive effects reported in both *in vitro* culture models and animal studies, cannabidiol (CBD) has emerged as a potential anticancer medication (Kis et al., 2019; Velasco et al., 2012; Wang and Multhoff, 2021). CBD application resulted in apoptotic cell death in the A549 and H460 lung cancer cell lines (Ramer et al., 2013) and reduced cell division and migration in diverse types of breast cancer cell lines (Elbaz et al., 2015). In recent years, increasing preclinical and translational studies have investigated the anti-proliferative, pro-apoptotic, and anti-invasive effects of cannabidiol (CBD) in glioma and other CNS malignancies. Recent systematic and translational reviews (Feng et al., 2024; Javid et al., 2025) highlight the growing interest in CBD as a potential adjunct or alternative therapeutic agent in glioma management. CBD and THC synergistically reduce glioma cell proliferation and modulate cell-cycle progression (Marcu et al., 2010), while recent studies in the SH-SY5Y neuroblastoma cell line have indicated that extracts isolated from *Cannabis sativa* strains with high CBD content can lead to apoptotic cell death (Sanchez-Sanchez et al., 2023) and modulate the expression of genes involved in cell adhesion and mitochondrial activities (Abyadeh et al., 2023). Although accumulating evidence supports the anti-tumor potential of CBD, recent analyses emphasize that its dose-dependent effects and cell-type specificity remain incompletely characterized, particularly in the context of balancing tumor inhibition with neuronal safety (Javid et al., 2025).

Despite increasing mechanistic insights, few studies have directly compared tumor-derived and non-malignant neuronal cell models under identical experimental conditions. Moreover, global proteomic responses to sublethal CBD exposure in glioma cells remain insufficiently characterized. Therefore, the present study aimed to compare the CBD-treated neuronal and glial tumor cell lines in terms of their cell viability, clonal formation, migration, and apoptotic morphology. In addition, we performed label-free quantitative proteomic analysis in C6 glioma cells exposed to sublethal CBD concentrations to identify early molecular alterations associated with cellular stress and anti-migratory effects. An immortalized hippocampal neuronal cell line (HT22) was included to comparatively evaluate the potential cytotoxic effects of CBD on non-malignant neuronal cells. This approach allows comparative evaluation of tumor and neuronal sensitivity to CBD exposure and provides preliminary insight into whether a potential therapeutic window may exist. The findings of this study can provide novel insights into the safe and effective administration of CBD in *in vivo* models and clinical trials.

## 2 Materials and methods

### 2.1 Cell culture and cannabidiol

The glioma cell line C6 derived from Wistar rat brain (CCL107) and human neuroblastoma cell line SH-SY5Y (CRL-2266) were acquired from the American Type Culture Collection (ATCC) and chosen in this study as archetypal tumor cell lines of CNS. The immortalized neuronal cell line HT22 (SCC129) derived from the mouse hippocampus was sourced from EMD Millipore and was used as a model of immortalized neurons. Dulbecco’s modified Eagle’s medium (DMEM) (ATCC, 30-2002) was used as a culture medium for maintaining the C6 and HT22 cell lines, while DMEM/F-12 Ham mixture (Millipore, DF-042-B) was used for maintaining SH-SY5Y cells. Both types of culture media were completed with 10% fetal bovine serum (FBS) (Sigma-Aldrich, F7524) and 10 mL/L of penicillin/streptomycin (Sigma-Aldrich, P4333). Cultures were maintained in a humidified incubator at the conditions of 5% CO2 at 37 °C, and the culture media were replaced with new media every 2-3 days. Dr. CBD Co., Ltd. (Bangkok, Thailand) provided the powdered CBD that we reconstituted and used in all experiments. We dissolved powdered CBD in dimethyl sulfoxide (DMSO) to create a 3 mg/mL stock solution, which we subsequently aliquoted, kept at −20 °C, and protected from light until use. In all experiments, vehicle control groups received an equivalent concentration of DMSO (0.1% v/v), which did not affect cellular outcomes.

### 2.2 MTT cell viability assay

The colorimetric MTT assay was performed to evaluate each cell line’s viability depending on the metabolic activity of mitochondrial oxidoreductase enzymes after treatment with CBD. Cells were seeded at a density of 1 × 10^4^ cells/well in a 96-well plate 24 h before assay. Five diluted concentrations (0.5, 1.0, 2.5, 5.0, and 10 μg/mL) of CBD in complete culture medium were added to the cultured cells in the treatment groups. After 24 h of incubation, the CBD-treated culture media were substituted with 1 mg/mL of MTT (Invitrogen, M6494) solution dissolved in complete media, before leaving in an incubator for reaction for 2 h. After dissolving the purple formazan precipitations in 50 µL of DMSO, a microplate reader (Thermo Fisher Scientific) was used to measure the absorbance of each culture well at 570 nm. Cell viability was expressed as a percentage relative to the vehicle control group. Concentration–response curves were generated by plotting percentage cell viability against the logarithm of CBD concentration. Nonlinear regression analysis was performed using a four-parameter logistic model (log [inhibitor] vs. normalized response–variable slope) in GraphPad Prism version 8.0 (GraphPad Software, San Diego, CA, USA). The half-maximal inhibitory concentration (IC_50_) values were calculated automatically from the fitted curves.

### 2.3 Clonogenic assay

We assessed the capability of studied CNS tumor cultures to establish colonies from a small initial population, simulating the growth of metastatic cancer cells, using a clonogenic assay (Franken et al., 2006). The starting cell population for both C6 and HT22 cultures was 500 cells per well, and seeded in the 6-well plate, while the starting cell population for SH-SY5Y culture was 2,000 cells per well, and seeded in a 12-well plate as optimized in our recent study (Viwatpinyo et al., 2023). The cells were incubated for 3-4 h until they were attached to the culture plate surfaces. Then, culture media were switched to CBD-added media (0.5, 1.0, 2.5, 5.0, and 10 μg/mL of CBD in complete media). After a 24-h incubation period, CBD-treated media were removed and the cells were washed using phosphate-buffered saline (PBS), and cells were continuously maintained with complete media with replacement every 2 days. Colony formation for the HT22 and C6 cell population was monitored for 6 days prior to being fixed with ice-cold methanol and stained with 0.5% w/v crystal violet for 30 min before being mildly washed with tap water. Because of their slower growth, SH-SY5Y colony formation was observed for 20 days after CBD treatment. On day 21, the cells were fixed and stained. The colonies stained with crystal violet on the culture plates were allowed to air dry for at least 24 h before being photographed using a stereomicroscope. The ImageJ software was used to analyze and calculate the colony area percentage in each well.

### 2.4 Scratch wound healing assay

The scratch assay was aimed to assess if CBD affects migration in tested tumor cells similar to our previous research (Viwatpinyo et al., 2023). All three cell lines were seeded at a density of 1 × 10^6^ cells/well in 6-well plates. After reaching full confluency, the cell cultures were scratched using an SPLScarTM cell scratcher (SPL Life Science Co., Ltd., Kyonggi-do, South Korea) creating a vertical 200 mm gap in the center of the culture plates. Thereafter, the cultures were cleansed from cell debris with PBS, then CBD-treated media (0.5, 1.0, 2.5, and 5.0 μg/mL CBD) were added. The scratch wounds were photographed immediately and after 24 h of incubation. Non-migrated areas at test endpoints were compared with scratched areas at test beginnings using ImageJ and the Wound Healing Size Tool plugin, as recommended by the plugin’s developers (Suarez-Arnedo et al., 2020).

### 2.5 Hoechst 33342/propidium iodide staining assay

To evaluate morphological features associated with apoptotic cell death, we performed Hoechst 33342 and propidium iodide (PI) double staining using a previously described protocol (Viwatpinyo et al., 2023). Hoechst 33342 was used to visualize nuclear morphology, including chromatin condensation and fragmentation, while PI was used to assess loss of membrane integrity. The cells were seeded at a density of 2 × 10^4^ cells/well in 12-well plates and incubated for at least 24 h. Then, media were substituted with CBD-treated media as mentioned in previous methods, and cultures were further incubated for an additional 24 h. At the endpoint of this experiment, the cells were stained for 30 min with a combination of PBS-diluted Hoechst 33342 (Abcam, ab228551) and propidium iodide (Sigma-Aldrich, P4170), and nuclei in cultures were subsequently digitally captured using an inverted fluorescence microscope (Zeiss AxioVert A1).

### 2.6 Proteomics analysis

#### 2.6.1 Proteomics sample preparation

For proteomic analysis in C6 glioma cells, three experimental groups were established: Vehicle control, 1 μg/mL CBD (sublethal concentration), and 2.5 μg/mL CBD (moderate sublethal concentration). These concentrations were selected based on MTT results to represent early molecular stress conditions without extensive cell death. Each experimental condition was performed in three independent biological replicates. Protein cell pellets were processed using the same method as described previously (Suwanchaikasem et al., 2024). Protein extracts were desalted and buffer-exchanged into 50 mM ammonium bicarbonate using Bio-Gel P6 spin columns (Bio-Rad, Hercules, CA, USA). The mixtures were centrifuged at 14,000 rpm for 5 min, and the resulting supernatants were collected. Protein concentrations were quantified using the Bradford assay and adjusted to 1.0 mg/mL. Each sample was then reduced with 100 mM dithiothreitol at 65 °C for 30 min, followed by alkylation with 500 mM iodoacetamide for 20 min at room temperature in the dark. Enzymatic digestion was performed by adding 2.5 µL of 0.1 mg/mL trypsin and incubating the reaction at 37 °C overnight. The digestion was stopped with 10% formic acid, and the mixture was centrifuged again at 14,000 rpm for 10 min. The clarified supernatant was finally transferred to polypropylene vials for subsequent LC-MS/MS analysis.

#### 2.6.2 LC-MS/MS proteomics acquisition

Proteomic analyses were performed using an Agilent 1290 Infinity II liquid chromatography system coupled to a 6545XT quadrupole time-of-flight mass spectrometer (Agilent Technologies, USA) equipped with an Agilent AdvanceBio Peptide Mapping column (120 Å, 2.1 × 150 mm, 2.7 µm). A 20-µL aliquot of each sample was injected for analysis. The mobile phases consisted of 0.1% formic acid in water (phase A) and acetonitrile (phase B). Data acquisition was conducted in positive ionization mode. All other LC and MS parameters were set according to conditions described in previously published protocols (Suwanchaikasem et al., 2024; Daduang et al., 2025).

#### 2.6.3 Proteomics data processing

Data was processed as described in the previous publication (Daduang et al., 2025). Raw data files generated in Agilent. d format were converted to. mzML and. mzXML formats prior to analysis with MaxQuant software (version 2.4). Trypsin was specified as the proteolytic enzyme, permitting up to two missed cleavages. Methionine oxidation and N-terminal acetylation were designated as variable modifications, whereas cysteine carbamidomethylation was defined as a fixed modification. Default settings optimized for Agilent Q-TOF instruments were applied. The false discovery rate (FDR) for both protein and peptide identification was maintained at 1%. Label-free quantification (LFQ) was performed with the minimum ratio count set to one peptide. Protein identification was conducted using the *Rattus norvegicus* reference proteome (taxonomy ID: 10116) obtained from the UniProt database. Entries identified by site or as reverse sequences were excluded. LFQ intensity values were subsequently processed using MetaboAnalyst version 6.0 (https://www.metaboanalyst.ca/), where data were log_10_-transformed and mean-centered. Differential protein expression analysis was performed using log_2_-transformed LFQ intensity values. Fold change (FC) was calculated by comparing CBD-treated groups with the untreated control group. Proteins were classified as upregulated when exhibiting positive log_2_FC values and downregulated when exhibiting negative log_2_FC values. Statistical significance was determined using Student’s t-test, and proteins with p < 0.05 were considered significantly regulated. To control for multiple hypothesis testing, false discovery rate (FDR) correction was applied, and proteins with FDR <0.05 were retained for further biological interpretation. Differentially expressed proteins (DEPs) identified through statistical analysis were subjected to Gene Ontology (GO) and pathway enrichment analyses using the DAVID bioinformatics resource (https://david.ncifcrf.gov/) (Huang da et al., 2009a; Huang da et al., 2009b).

### 2.7 Statistical analysis

The assay data are displayed as the mean ± standard deviation of three separate experiments. To find statistical differences between the groups, Tukey’s multiple comparison test was employed in conjunction with One-way ANOVA. A value of p < 0.05 was considered statistically significant.

## 3 Results

**FIGURE 1:**
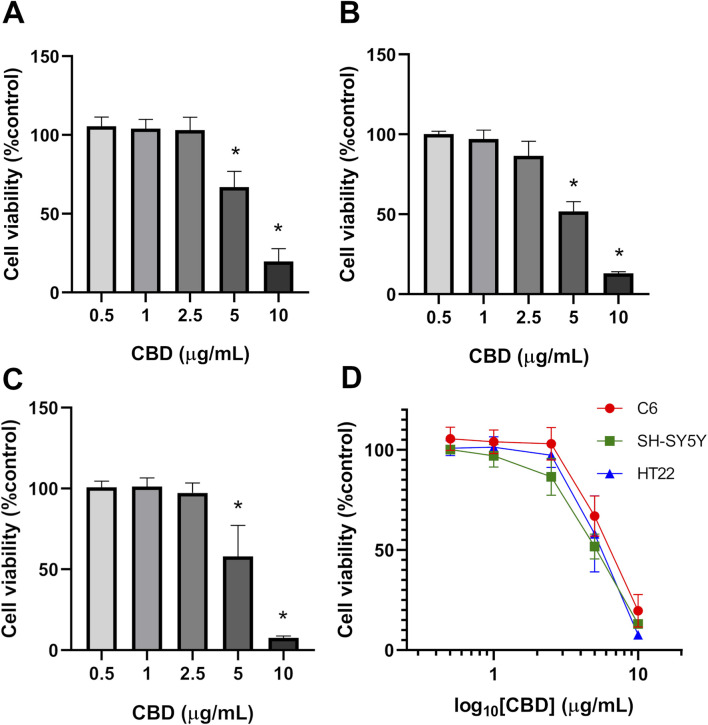
Effects of CBD on cell viability in **(A)** C6, **(B)** SH-SY5Y, and **(C)** HT22 cell lines. Bar graphs showing percentage cell viability assessed by MTT assay after 24 h treatment with CBD (0.5–10 μg/mL). Data are expressed as percentage relative to the vehicle control group (set as 100%). Values represent mean ± SD from three independent experiments (n = 3). Statistical analysis was performed using one-way ANOVA followed by Tukey’s multiple comparison test. *p < 0.05 vs. vehicle control. **(D)** Concentration–response curves of percentage cell viability against the logarithm of CBD concentration.

**FIGURE 2:**
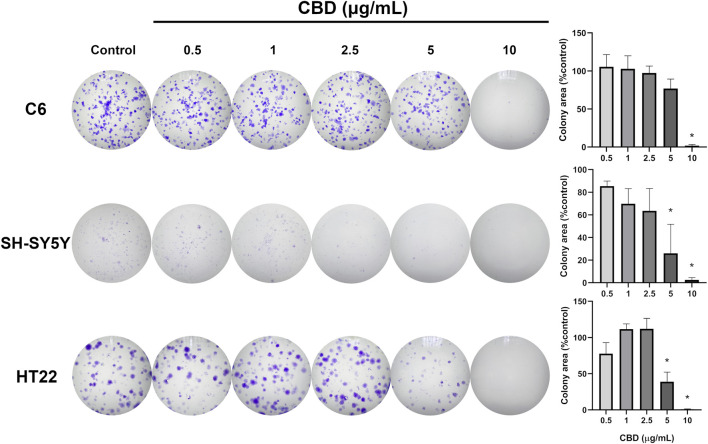
Images showing the crystal violet-stained colonies of C6 glioma, SH-SY5Y neuroblastoma, and HT22 hippocampal neuronal cells. Bar graphs on the right represent the percentages of calculated colony areas in each treatment group compared with the control group. Statistical analysis was performed using one-way ANOVA followed by Tukey’s multiple comparison test. Data are expressed as the mean ± standard deviation from three independent experiments. An asterisk (*) indicates a significant difference from the control group at p < 0.05.

Results showed that CBD at 5 and 10 μg/mL significantly reduced the cell viability of all three cell lines (Figure 1). Based on nonlinear regression analysis of concentration–response curves, the IC_50_ values of CBD for C6, SH-SY5Y, and HT22 cells were 6.949 μg/mL (22.097 μM), 5.851 μg/mL (18.616 μM), and 5.966 μg/mL (18.972 μM), respectively. These results imply that the cytotoxic effect of CBD was most potent on SH-SY5Y neuroblastoma cells, followed by HT22 and C6 cells. We found that CBD suppressed the proliferation of the tested tumor cell lines and decreased colony formation. Compared with the untreated control group (Figure 2), CBD treatment significantly impaired clonogenic capacity in all examined cell lines. In C6 glioma cells, exposure to 10 μg/mL CBD almost completely abolished colony formation. Similarly, SH-SY5Y neuroblastoma cells exhibited a pronounced reduction in colony growth at 5 and 10 μg/mL. In HT22 neuronal cultures, CBD induced a clear concentration-dependent decline in clonogenic potential. Lower concentrations (0.5–2.5 μg/mL) produced modest reductions in colony area, whereas 5 μg/mL significantly suppressed colony formation. At 10 μg/mL, colony growth was nearly eliminated, with only sparse and markedly diminished colonies observed. Quantitative analysis of colony area percentage confirmed statistically significant reductions compared with the vehicle control group (p < 0.05).

**FIGURE 3:**
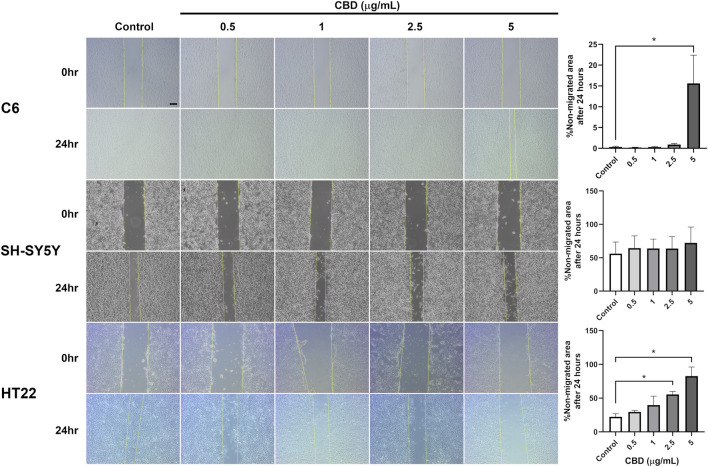
Photomicrographs of representative scratched confluence cultures of C6, SH-SY5Y, and HT22 cells at 0 and 24 h after treatment with 0–5 μg/mL CBD. The scale bar represents 100 μm. Bar graphs on the right represent the percentages of calculated non-migrated areas in each treatment group compared with the control group after 24 h. Statistical analysis was performed using one-way ANOVA followed by Tukey’s multiple comparison test. Data are expressed as the mean ± standard deviation from three independent experiments. An asterisk (*) indicates a significant difference from the control group at p < 0.05.

In CBD-treated C6 and HT22 cell cultures, migration into the scratched area was attenuated in a concentration-dependent manner; however, this inhibitory effect was not observed in CBD-treated SH-SY5Y cell culture (Figure 3). C6 cell migration was repressed when exposed to 5 μg/mL CBD for 24 h, with up to 15% of the scratched area remaining non-migrated at the experiment’s endpoint. Furthermore, both 2.5 and 5 μg/mL CBD significantly reduced HT22 cell migration compared with the vehicle control group.

**FIGURE 4:**
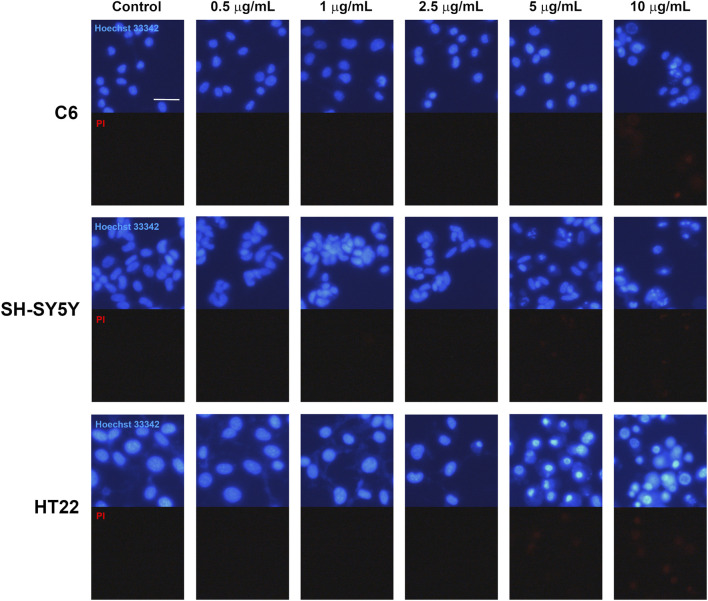
Photomicrographs of representative C6, SH-SY5Y, and HT22 cultures stained with Hoechst 33342 and PI after 0–10 μg/mL CBD treatment for 24 h. Bright blue signals indicate nuclear condensation and fragmentation in cultures treated with 5 and 10 μg/mL of CBD. Intracellular red PI signals were observed in cultures treated with 10 μg/mL of CBD. The scale bar represents 10 μm.

Hoechst 33342 and PI double staining was performed to evaluate morphological features associated with CBD-induced cell death in the tested cell lines (Figure 4). At CBD concentrations below 5 μg/mL, Hoechst 33342 fluorescence appeared evenly distributed within the nuclei of both vehicle-control and treated groups across all three cell lines, indicating preserved nuclear morphology.

In C6 glioma cells treated with 10 μg/mL CBD, marked nuclear condensation and fragmentation were observed, accompanied by increased PI-positive staining, indicating loss of plasma membrane integrity. Similarly, SH-SY5Y neuroblastoma and HT22 neuronal cells exposed to 5 and 10 μg/mL CBD exhibited numerous cells displaying condensed and fragmented nuclei together with prominent PI fluorescence. These morphological features are consistent with apoptotic cell death accompanied by membrane compromise. Collectively, these findings support that CBD treatment induces cell death with apoptotic characteristics in all examined cell lines, in agreement with the reductions in cell viability observed in the MTT assay.

**FIGURE 5:**
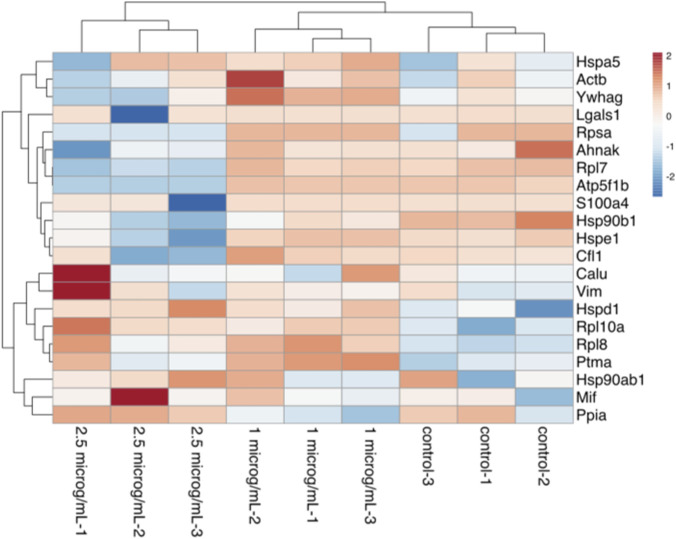
Hierarchical clustering heatmap of significantly differentially expressed proteins (n = 21; p < 0.05) in C6 glioma cells following treatment with 1 and 2.5 μg/mL CBD. Log_2_-transformed LFQ intensities were mean-centered and scaled by row. Red indicates relative upregulation and blue indicates relative downregulation compared with control samples. Distinct clustering of control and CBD-treated groups demonstrates reproducible proteomic alterations induced by CBD exposure.

To investigate early molecular alterations preceding extensive cytotoxicity, we selected sublethal CBD concentrations (1 and 2.5 μg/mL) for proteomic analysis. These concentrations produced minimal to moderate reductions in cell viability but did not induce extensive cell death, thereby allowing identification of primary stress-response pathways rather than secondary effects associated with advanced apoptosis. Using LC/MS-based analysis, a total of 116 protein groups were detected across all experimental groups, with 21 protein groups showing significant differences (p-value <0.05). Proteomic analysis of C6 cells treated with 1 μg/mL CBD revealed distinct alterations in protein expression compared to the untreated control (Table 1). Several proteins were upregulated in the CBD-treated group, including large ribosomal subunit proteins (uL30/Rpl7, uL1/Rpl10a, uL2/Rpl8), molecular chaperones (Hsp90b1, Hspe1, Hspd1), calumenin (Calu), and macrophage migration inhibitory factor (Mif), all with highly significant changes (p < 10^−10^, FDR <10^−9^). In addition, proteins such as prothymosin alpha (Ptma), cofilin-1 (Cfl1), and peptidyl-prolyl cis–trans isomerase A (Ppia) were increased in their expression in the CBD group, although with lower statistical significance (FDR 0.02–0.03). Several proteins were significantly downregulated in the C6 cells treated with 2.5 μg/mL CBD (Table 2), including the endoplasmic reticulum chaperone BiP (Hspa5), ATP synthase subunit beta (Atp5f1b), protein S100-A4 (S100a4), and AHNAK nucleoprotein (Ahnak), with highly significant differences (p < 10^−9^, FDR <10^−8^). The expressions of the core cytoskeletal protein actin (Actb) and regulatory protein 14-3-3 protein gamma (Ywhag) were also decreased following CBD exposure (p < 0.001, FDR <0.002). In contrast, prothymosin alpha (Ptma), cofilin-1 (Cfl1), and histone H1.4 (H1-4) displayed moderate increases in abundance, while galectin-1 (Lgals1), vimentin (Vim), and small ribosomal subunit protein uS2 (Rpsa) exhibited reduced expression, albeit with lower statistical significance (FDR 0.02–0.07). A hierarchical clustering heatmap of significant DEPs (n = 21; p < 0.05) demonstrated clear separation between control and CBD-treated groups (Figure 5). Log_2_-transformed LFQ values revealed consistent protein expression patterns across replicates and dose-dependent alterations induced by CBD treatment. To provide a clearer functional interpretation of these findings, selected DEPs were further categorized according to their biological roles in proteostasis, mitochondrial function, cytoskeletal organization, and apoptosis-related signaling pathways (Table 3). This summary highlights key regulatory nodes potentially involved in CBD-induced cellular stress responses and apoptotic mechanisms in C6 glioma cells.

**TABLE 1:** Differentially expressed proteins identified in C6 rat glioma cells following treatment with 1 μg/mL cannabidiol (CBD). Proteins are listed with UniProt accession ID, gene and protein names, average LFQ intensities in control and CBD-treated groups, p values, –log10(p), and FDR values. LFQ intensity values of 0.00 indicate that the protein was not detected or was below the quantifiable detection limit under that condition.

UniProt accession ID	Protein name	Gene name	Average LFQ intensity		p value	-log10(P)	FDR
			Control	1 μg/mL CBD			
P05426	Large ribosomal subunit protein uL30	Rpl7	0.00	7,296.33	1.5714 × 10^−15^	14.804	1.1157 × 10^−13^
Q66HD0	Endoplasmin	Hsp90b1	0.00	8,154.07	9.9864 × 10^−13^	12.001	3.5452 × 10^−11^
P26772	10 kDa heat shock protein, mitochondrial	Hspe1	0.00	8,816.97	2.7603 × 10^−12^	11.559	4.8995 × 10^−11^
P63039	60 kDa heat shock protein, mitochondrial	Hspd1	0.00	9,590.20	1.676 × 10^−11^	10.776	2.3799 × 10^−10^
A0A8I6A8Z7	Calumenin	Calu	0.00	7,754.53	2.745 × 10^−11^	10.561	3.2482 × 10^−10^
P62907	Large ribosomal subunit protein uL1	Rpl10a	0.00	9,295.30	5.0127 × 10^−11^	10.3	5.0843 × 10^−10^
P30904	Macrophage migration inhibitory factor	Mif	0.00	7,965.70	7.705 × 10^−11^	10.113	6.8382 × 10^−10^
P62919	Large ribosomal subunit protein uL2	Rpl8	0.00	10,639.93	1.0648 × 10^−9^	8.9727	7.56 × 10^−9^
P06302	Prothymosin alpha	Ptma	13,626.00	17,171.67	0.0052078	2.2833	0.02465
P45592	Cofilin-1	Cfl1	40,918.67	48,545.33	0.0056784	2.2458	0.025198
P10111	Peptidyl-prolyl cis-trans isomerase A	Ppia	42,437.33	48,005.67	0.0094296	2.0255	0.033475
A0A8I5Y6H8	Filamin A	Flna	0.00	8,994.93	0.038778	1.4114	0.083433
P16617	Phosphoglycerate kinase 1	Pgk1	0.00	10,418.33	0.040204	1.3957	0.083955

**TABLE 2:** Differentially expressed proteins identified in C6 rat glioma cells following treatment with 2.5 μg/mL cannabidiol (CBD). Proteins are listed with UniProt accession ID, gene and protein names, average LFQ intensities in control and CBD-treated groups, p values, –log10(p), and FDR values. LFQ intensity values of 0.00 indicate that the protein was not detected or was below the quantifiable detection limit under that condition.

UniProt accession ID	Protein name	Gene name	Average LFQ intensity		p value	-log10(P)	FDR
			Control	2.5 μg/mL CBD			
P06761	Endoplasmic reticulum chaperone BiP	Hspa5	12,217.00	0.00	2.5945 × 10^−12^	11.586	4.8995 × 10^−11^
P05942	Protein S100-A4	S100a4	9,108.07	0.00	5.2065 × 10^−10^	9.2835	4.1073 × 10^−9^
P10719	ATP synthase subunit beta, mitochondrial	Atp5f1b	13,535.00	0.00	1.5372 × 10^−9^	8.8133	9.9217 × 10^−9^
A0A0G2JUA5	AHNAK nucleoprotein	Ahnak	14,514.53	0.00	1.0671 × 10^−6^	5.9718	6.3135 × 10^−6^
P60711	Actin, cytoplasmic 1	Actb	164,303.33	143,013.33	0.000010076	4.9967	0.000055032
P61983	14-3-3 protein gamma	Ywhag	20,157.67	16,493.67	0.00028946	3.5384	0.001468
P06302	Prothymosin alpha	Ptma	13,626.00	17,951.33	0.0052078	2.2833	0.02465
P45592	Cofilin-1	Cfl1	40,918.67	46,056.67	0.0056784	2.2458	0.025198
P11762	Galectin-1	Lgals1	67,781.67	61,162.67	0.0086312	2.0639	0.033475
P38983	Small ribosomal subunit protein uS2	Rpsa	18,096.00	16,493.33	0.0089739	2.047	0.033475
P31000	Vimentin	Vim	25,760.00	19,419.67	0.0127	1.8962	0.042936
P15865	Histone H1.4	H1-4	18,734.00	21,903.00	0.02698	1.569	0.070472

**TABLE 3:** Functional summary of selected differentially expressed proteins identified in C6 rat glioma cells following treatment with cannabidiol (CBD). Proteins are grouped according to direction of regulation at 1 and 2.5 μg/mL CBD. Selection was based on statistical significance (p < 0.05) and biological relevance to endoplasmic reticulum stress, mitochondrial function, cytoskeletal organization, and apoptosis-related pathways.

Gene	Protein	Regulation	Biological function	Relevance to apoptosis/Stress
Hsp90b1	Endoplasmin (GRP94)	↑ (1 μg/mL)	ER chaperone protein folding	ER stress response modulation
Hspd1	Hsp60	↑ (1 μg/mL)	Mitochondrial chaperone	Mitochondrial protein quality control
Ptma	Prothymosin alpha	↑ (1 μg/mL)	Chromatin remodeling	Nuclear apoptosis regulation
Mif	Macrophage migration inhibitory factor	↑ (1 μg/mL)	Inflammatory mediator	Stress response signaling
Cfl1	Cofilin-1	↑ (1 and 2.5 μg/mL)	Actin depolymerization	Cytoskeletal remodeling during apoptosis
Hspa5	BiP (GRP78)	↓ (2.5 μg/mL)	ER stress sensor	Reduced adaptive UPR signaling
Atp5f1b	ATP synthase β	↓ (2.5 μg/mL)	Mitochondrial ATP production	Impaired mitochondrial function
Actb	β-Actin	↓ (2.5 μg/mL)	Cytoskeletal integrity	Migration and apoptotic morphology
S100a4	S100-A4	↓ (2.5 μg/mL)	Cell motility regulator	Migration inhibition
Ywhag	14-3-3γ	↓ (2.5 μg/mL)	Cell survival signaling	Apoptosis regulatory pathways

## 4 Discussion

In this study, we comparatively evaluated the cytotoxic and anti-migratory effects of CBD in CNS tumor cell lines (C6 and SH-SY5Y) and in immortalized neuronal HT22 cells to explore their relative sensitivity to CBD exposure. Our findings demonstrate that CBD exerts dose-dependent anti-proliferative effects across all three cell lines, with tumor cells and neuronal cells exhibiting comparable sensitivity at higher concentrations. These results highlight the complexity of defining a therapeutic window for CBD in CNS applications. The IC_50_ values of CBD against C6, SH-SY5Y, and HT22 cells were 22.097, 18.616, and 18.972 μM, respectively. Similar results have been observed in previous studies. For example, the IC_50_ value of CBD against U87 and U373 glioma cells is 26.2 and 24.1 μM, respectively (Massi et al., 2004). Another study investigating CBD cytotoxicity in the primary glioblastoma cell line MZC found that the IC_50_ value after 24 h of CBD treatment is 33.2 μM (Nabissi et al., 2013). A recent study has reported that isolated CBD is cytotoxic to SH-SY5Y neuroblastoma, with an IC_50_ of approximately 40 μM (Urasaki et al., 2020). This difference in IC_50_ values may be due to the different extraction and purification methods used by these studies. Recent translational analyses further support the anti-proliferative potential of CBD in glioma models., which concluded that CBD induces glioma cell death through mechanisms involving oxidative stress, mitochondrial dysfunction, ER stress signaling, and autophagy modulation (Feng et al., 2024). Our observed IC_50_ values in C6 cells fall within the range reported for other glioma models and are consistent with these proposed mechanisms, particularly given the proteomic alterations observed in mitochondrial and chaperone-related proteins. Although reports on CBD cytotoxicity in HT22 neuronal cells are limited, a 2023 study found that 24-h treatment with 100 μM CBD can reduce cell viability to 16.9% of the untreated group (Drummond-Main et al., 2023). Another recent study found that the IC_50_ value of CBD against rat primary hippocampal neurons is 9.85 μM (Kim et al., 2021), suggesting that the primary cultures of normal neurons are more sensitive to CBD than immortalized neuronal cultures. The apparent sensitivity of HT22 neuronal cells at higher CBD concentrations should be interpreted considering previous studies demonstrating context-dependent neuronal responses. Previous research reported that CBD exposure during neuronal differentiation modulates redox balance and may alter susceptibility to oxidative stress (Schonhofen et al., 2015). These findings suggest that neuronal responses to CBD are influenced by developmental state, oxidative status, and exposure conditions. Therefore, the cytotoxic effects observed in immortalized HT22 cells at higher concentrations do not necessarily contradict reports of neuroprotection at lower doses, but rather highlight the concentration- and context-dependent nature of CBD action.

Hoechst 33342/PI staining revealed that 10 μg/mL CBD triggered apparent nuclear morphology that concurred with apoptotic cell death in all three cell lines. Additionally, PI staining in cells exposed to this concentration of CBD suggested a loss of plasma membrane integrity in dead cells due to apoptosis. CBD may induce apoptosis in glioma cells via increased oxidative stress, glutathione depletion, and subsequent caspase-9, -8, and -3 activation (Massi et al., 2004; Massi et al., 2006). Another study found that a THC and CBD combination can synergistically increase reactive oxygen species and significantly elevate apoptotic glioma cells (Marcu et al., 2010). The involvement of cannabinoid-induced oxidative stress that leads to tumor apoptosis has also been demonstrated in several neoplastic cell lines, including PC-12 pheochromocytoma (Sarker et al., 2000), EL-4 thymoma (Lee et al., 2008), and MDA-MB-231 human breast cancer cells (Ligresti et al., 2006), Increased production of intracellular sphingolipid ceramide due to cannabinoids might be another possible mechanism. Ellert-Miklaszewska et al. (2013) reviewed that ceramides suppress pro-survival pathways in glioma cells, such as the PI3K/Akt and Raf/MEK/ERK pathways, resulting in apoptosis. Interestingly, THC has been found to promote dihydroceramide accumulation followed by lysosomal membrane permeabilization, cathepsin release, and apoptotic cell death (Hernandez-Tiedra et al., 2016). Thus, CBD may induce apoptosis in glioma cell cultures via multiple mechanisms, offering therapeutic potential for glioma and other malignancies. Although Hoechst 33342/PI staining enables visualization of apoptotic morphology and membrane integrity changes, it does not allow precise staging of apoptosis. Quantitative methods such as Annexin V/PI flow cytometry would provide more definitive characterization.

The clonogenic assay further supports the anti-proliferative effect of CBD by demonstrating impaired long-term reproductive capacity following short-term exposure. Unlike the MTT assay, which reflects short-term metabolic activity, the clonogenic assay evaluates the ability of single cells to survive treatment and re-establish colonies over time. In our study, higher concentrations of CBD markedly reduced colony formation in C6 and SH-SY5Y tumor cells, indicating sustained suppression of proliferative potential beyond acute cytotoxicity. The apparent discrepancy between MTT and clonogenic results in SH-SY5Y cells may reflect fundamental differences between these assays. While the MTT assay measures short-term metabolic activity following 24 h exposure, the clonogenic assay evaluates long-term reproductive survival and the ability of single cells to re-enter the cell cycle and form colonies over an extended period. These endpoints are not always directly proportional. Moreover, clonogenic assays require low initial seeding density, and plating efficiency may vary between cell lines, particularly in neuroblastoma-derived cells, which can exhibit heterogeneous growth characteristics. Therefore, differences observed between metabolic viability and long-term colony formation likely reflect biological differences in proliferative recovery rather than experimental error. Notably, HT22 neuronal cells also exhibited a concentration-dependent decline in clonogenic capacity, with near-complete inhibition at 10 μg/mL. These findings suggest that prolonged proliferative impairment occurs in both malignant and non-malignant neuronal models at higher concentrations, reinforcing the narrow separation between anti-tumor efficacy and neuronal toxicity observed in this study. A limitation of the clonogenic analysis is that colony growth was quantified based on colony area percentage rather than counting individual cells per colony. Due to fixation and crystal violet staining procedures, individual cell boundaries could not be reliably distinguished. While colony area is widely accepted as a surrogate indicator of proliferative potential, future studies employing automated colony counting or live-cell imaging may provide more refined assessment of cellular reproductive capacity.

The migration of C6 glioma cells was significantly suppressed after CBD exposure at 5 μg/mL for 24 h. Our findings regarding the anti-migratory effect of CBD in C6 glioma cells are consistent with previous reports demonstrating that specific compositions of *Cannabis sativa* compounds significantly inhibited migration and colony formation of human glioblastoma A127 cells using both scratch wound healing and transwell assays (Peeri et al., 2021). Their results indicated that cannabinoid exposure reduces cellular motility and proliferative capacity, supporting the concept that cannabinoids interfere with cytoskeletal dynamics and invasive behavior. The suppression of migration observed in our C6 model further corroborates these findings and suggests that CBD-mediated inhibition of motility may represent a conserved response across different glioma subtypes. Moreover, a previous study investigating the migration of CBD-treated U87 glioma cells in a Boyden chamber (Vaccani et al., 2005) suggested that the anti-migratory effect of CBD may not depend on cannabinoid receptors. A 72-h exposure to 1 μM CBD was found to significantly reduce invasiveness of U251 glioma and primary glioblastoma neurosphere cells, which is correlated with the downregulation of Id-1, a transcription factor linked with tumor invasiveness (Soroceanu et al., 2013) and is regulated by several pathways, including the TGF-β and MAPK/Egr1 pathways (Zhao et al., 2020), which may be affected by CBD. We did not observe a significant reduction in cell migration for SH-SY5Y neuroblastoma after 24 h of treatment with 5 μg/mL CBD, which is different from prior findings (Alharris et al., 2019). This may stem from the different models of cell migration used between our study and the previous one. The previous study’s model, using transwell chambers and Matrigel-coated inserts, allowed the cells to migrate more freely, potentially offering a more realistic simulation of neuroblastoma migration and invasion in tissues. Nevertheless, recent migration studies have employed scratch assays similar to our study (de Moura Escobar et al., 2019; Guo et al., 2021). To evaluate these variations and the underlying processes by which CBD influences neuroblastoma migration, a more in-depth investigation is necessary.

**FIGURE 6:**
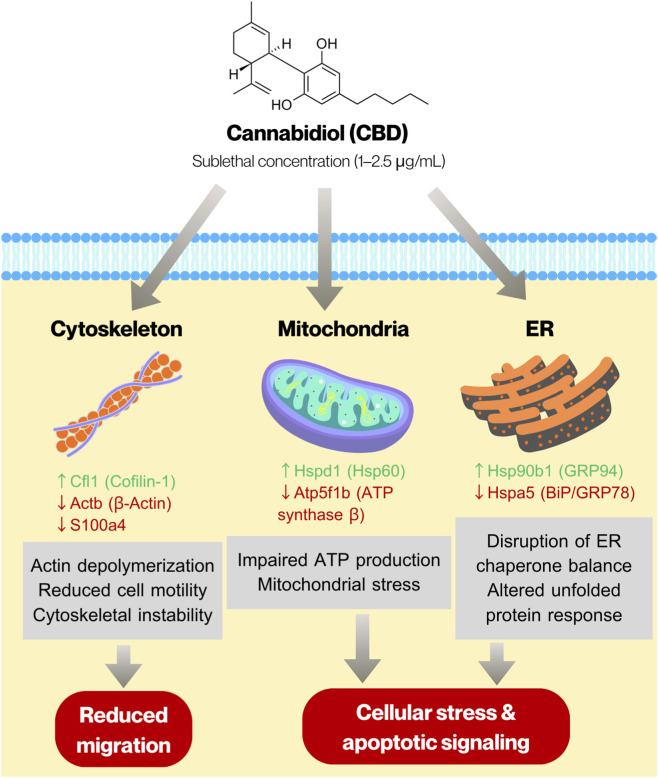
Proposed mechanism of cannabidiol-induced cytotoxicity and anti-migratory effects in C6 glioma cells. CBD treatment alters proteostasis by modulating ER chaperones (Hsp90b1, Hspa5), disrupts mitochondrial bioenergetics through downregulation of ATP synthase (Atp5f1b), and induces cytoskeletal remodeling via regulation of actin-associated proteins (Actb, Cfl1, S100a4). These coordinated changes may contribute to apoptosis and suppressed cell migration.

We also investigated the proteomic alterations in C6 glioma cells treated with 1 and 2.5 μg/mL CBD to identify proteins whose expression levels were affected by sublethal concentrations of CBD. Overall, treatment with 1 μg/mL CBD resulted in upregulation of proteins involved in protein synthesis and folding, whereas exposure to 2.5 μg/mL CBD led to significant downregulation of mitochondrial ATP synthase, molecular chaperones, and cytoskeletal proteins. These results suggest that CBD exposure triggers a robust proteostatic response, involving ribosomal and chaperone proteins, alongside modulators of cytoskeletal dynamics. At higher concentration, CBD disrupts chaperone-mediated protein folding and mitochondrial ATP synthesis, while simultaneously affecting cytoskeletal organization, ribosomal function, and nuclear structural proteins. Our proteomic findings are consistent with recent systematic analyses summarizing the molecular mechanisms underlying cannabinoid-mediated anti-glioma activity, which highlighted that cannabinoids modulate key pathways in glioma cells, including oxidative stress signaling, mitochondrial dysfunction, endoplasmic reticulum stress responses, and cytoskeletal remodeling (Javid et al., 2025). The observed downregulation of ATP synthase subunit Atp5f1b and ER chaperone Hspa5, along with altered expression of actin-associated proteins in our C6 model, aligns with these mechanistic themes. Importantly, our proteomic data extend these mechanistic insights by providing global protein-level evidence of coordinated alterations in proteostasis and bioenergetic regulation following sublethal CBD exposure. Our results are also comparable with a related proteomic study on CBD-treated SH-SY5Y neuroblastoma cells, in which treatment with 10 µM CBD (approximately 3.12 μg/mL) caused downregulation of several proteins associated with the mitochondrial ATP synthesis pathway (Abyadeh et al., 2023). Based on the integrated proteomic and functional findings, we constructed a schematic model to illustrate the potential mechanisms underlying CBD-induced cytotoxicity and anti-migratory effects in C6 glioma cells (Figure 6). The proposed model highlights coordinated alterations in endoplasmic reticulum proteostasis, mitochondrial bioenergetics, and cytoskeletal organization. Modulation of ER chaperones (Hsp90b1 and Hspa5), downregulation of mitochondrial ATP synthase (Atp5f1b), and regulation of actin-associated proteins (Actb, Cfl1, and S100a4) collectively suggest disruption of cellular stress adaptation and structural integrity. These molecular changes may contribute to the induction of apoptosis and reduced migratory capacity observed following CBD exposure.

Finally, our findings suggest that a high concentration of CBD may negatively affect the cellular processes of healthy neurons, as indicated by reduced cell viability, proliferation, and migration. Lower concentrations of CBD can be used for neuroprotection against insults. For example, 2.5 μM CBD treatment for 1 h protects rat primary cerebellar neurons from H_2_O_2_- and rotenone-induced neuronal injury (Echeverry et al., 2021), while a 1-h treatment of 1 or 10 μM CBD increases the viability of primary neuronal culture treated with methamphetamine (Shen et al., 2022). However, a systematic review in 2019 suggested that CBD is not risk-free, and its adverse effects should be considered (Huestis et al., 2019). The present findings illustrate a concentration-dependent duality of CBD action *in vitro*. Lower concentrations (≤2.5 μg/mL) exert limited anti-proliferative effects in tumor cells, while preserving relatively higher viability in HT22 neuronal cultures. In contrast, concentrations ≥5 μg/mL induce pronounced cytotoxicity in both tumor and neuronal cells. This overlapping sensitivity suggests that, under simplified *in vitro* conditions, the therapeutic window may be narrow. However, *in vivo* pharmacokinetics, blood–brain barrier distribution, tumor microenvironment differences, and differential oxidative stress status between malignant and non-malignant cells may influence selectivity. Therefore, our study does not define a clinically safe concentration, but rather highlights the complexity of balancing anti-tumor efficacy and neuronal safety. Investigation using stringent experimental paradigms and translational models is needed to pinpoint the processes underlying CBD-induced neurotoxicity and to provide guidance for its safe and effective therapeutic application.

In conclusion, our experiments revealed that 24-h treatment with CBD greater than 5 μg/mL can reduce the cell viability and clonogenicity of all studied cell cultures by stimulating apoptotic cell death, as revealed by nuclear condensation and fragmentation. Migration of C6 cells was suppressed by 5 μg/mL CBD, while that of HT22 by 2.5 μg/mL CBD and higher concentrations. Furthermore, sublethal CBD exposure provokes a proteotoxic response in glioma cells, characterized by impaired mitochondrial function and cytoskeletal integrity at higher concentrations. These alterations highlight mitochondrial and proteostatic disruption as key mechanisms contributing to CBD-induced cellular stress and toxicity. Importantly, our results indicate that the concentrations required to achieve substantial anti-tumor effects in C6 glioma and SH-SY5Y neuroblastoma cells overlap with those that impair viability and proliferation in HT22 neuronal cells. This suggests that the therapeutic window for CBD in CNS applications may be narrow under *in vitro* conditions. While lower concentrations have been reported to exert neuroprotective effects in certain experimental models, our findings indicate that these concentrations may not produce strong anti-tumor efficacy. Therefore, careful dose optimization and *in vivo* validation are necessary before clinical translation can be considered.

## Data Availability

The original contributions presented in the proteomic study are publicly available. This data can be found in ProteomeXchange with the accession number PXD076218 (http://proteomecentral.proteomexchange.org/ui?pxid=PXD076218), and in jPOST with the accession number JPST004518 (http://repository.jpostdb.org/entry/JPST004518).
